# Seroprevalence of hepatitis C in type 2 diabetes: evidence for a positive association

**DOI:** 10.1186/1743-422X-7-304

**Published:** 2010-11-05

**Authors:** Nauman A Jadoon, Mohammad A Shahzad, Rehan Yaqoob, Mansoor Hussain, Naseema Ali

**Affiliations:** 1 Department of Medicine, Nishtar Medical College Hospital, Multan, Pakistan

## Abstract

**Background:**

There is a growing body of literature on the relationship of Hepatitis C virus infection (HCV) and type 2 diabetes mellitus (T2DM). However, there are certain gaps in literature and the data is inconclusive. This study was, therefore, carried out to determine the prevalence of HCV infection in diabetic patients and to elucidate the presence of any possible relationship between HCV and T2DM in this region.

**Methods:**

Serologic testing for anti-HCV antibody was done on a sample of 3000 individuals with T2DM visiting Diabetes Clinic of Nishtar Medical College Hospital, Multan and 10,000 volunteer blood donors visiting blood bank of the same hospital during the study period using Accurate rapid immunochromatographic kits which was later confirmed by using Chemelex S.A third generation ELISA kit for positive cases. Data about various variables was collected from diabetic patients using a structured questionnaire after taking informed consent.

**Results:**

Prevalence rate of 13.7% for HCV infection was recorded among subjects having T2DM with seropositivity rate of 4.9% among the control group of volunteer blood donors without diabetes. The patients with T2DM were more likely to have HCV infection as compared to the control group (OR = 3.03, 95%CI = 2.64-3.48, p = 0.001). Diabetic patients with age above 55 years had higher prevalence rate as compared to younger individuals. Male patients had significantly high seropositivity as compared to female patients (15.3% vs. 12.4%, p = 0.02). Those with duration of diabetes 11 years and above and the ones with good glycemic control had higher seroprevalence rates of 18.2% and 18.7% respectively. There was no statistically significant difference among subjects when the distribution of HCV was studied on the basis of marital status, locality, or family history of diabetes.

**Conclusions:**

The results show that there is a strong association between HCV and T2DM in the region as evident from significantly higher prevalence of HCV infection in diabetics as compared to the control group in the present study.

## Background

Hepatitis C virus (HCV) infection is an important public health problem which currently affects more than 170 million people (about 3% of world population) out of which 55-80% have chronic infection [1]. It is a common cause of acute and chronic hepatitis accounting for about half of all the cases of CLD in USA [2]. The likelihood of chronicity after acute HCV infection is as high as 85% with chronic infection being common even in those having normal aminotransferase levels after the acute episode [3]. The progression to cirrhosis is up to 50% even in well compensated patients. The incidence of HCC in patients with cirrhosis ranges from 1-4%, most cases comprising of patients with HCV infection duration exceeding 30 years [3]. The severity and rate of progression depend on several disease related factors and various host related factors [3,4]. Infection with HCV has been shown to produce both hepatic and extrahepatic manifestations, the latter including insulin resistance, essential mixed cryoglobulinemia, glomerulonephritis, porphyria cutaneous tarda and benign monoclonal gammopathy [5,6]. A meta-analysis showed that HCV increases the risk of type 2 diabetes mellitus (T2DM) by 1.8 times in excess of that posed by relative degree of liver pathology [7].

The link between the HCV and diabetes was first reported by Allison et al. in 1994 and later explored by Simo and colleagues in 1996 [8,9]. The initial idea that patients with T2DM have more parenteral exposures because of use of finger stick devices and thus are at an increased risk of contacting blood borne infections such as HCV was disproved by a study from France in 1998 [10]. The epidemiological link between T2DM and HCV has been investigated from two perspectives. Various studies have shown high HCV seropositivity among patients with T2DM as compared to the control group, prevalence being two to seven times higher in the diabetic group [11-14]. However, other investagators performing did not find such an association of HCV with T2DM [15-17]. In addition, several studies have shown that HCV increases the risk of development of T2DM [7]. The mechanism of pathogenesis of diabetes in patients with HCV infection remains unclear though it has been implicated that insulin resistance plays an important role and is related to fibrosis score [18-20]. After controlling for potential confounders, Mehta et al. reported that HCV infected individuals were 3.77 times more likely (95%CI = 1.80-.87) to have T2DM as compared to those without the infection [21].

Pakistan is in the intermediate HCV prevalence area with approximately 10 million people infected predominately with genotype 3 based on an average prevalence rate of 6%. The prevalence ranges from 3-4% in volunteer blood donors according to a meta analysis. About 60-70% of the patients with CLD and half of the ones with HCC in the country have HCV infection [22]. Diabetes has become an important public health problem in Pakistan with 7.1 million diabetics in 2010 expected to rise to 13.8 million in 2030 when the country will rank fourth in terms of number of patients aged 20-79 with diabetes [23]

Although there is a growing body of literature on the link between T2DM and HCV, the studies are contradictory and the data is inconclusive [11,13-17,24-30]. Secondly, it is not known if diabetes is a risk factor for the development of HCV. The study will be valuable in this regard. Moreover, there is paucity of studies on the subject from Pakistan with no study being from this region to the best of our knowledge [12,31]. In addition, the previous studies from Pakistan employed a small sample size with no control group [12,31]. The present study will thus be important in elucidating any relationship between HCV and T2DM in the region. In addition, it is necessary to determine the prevalence of HCV among diabetics to increase awareness among general population and health care workers to prevent morbidity and increased costs associated with this infection in diabetes due to failure of treatment [24]. Since the prevalence of diabetes is on the rise and is complicated by coinfection with HCV, the determination of relationship becomes even more important in this scenario so that it can be effectively managed [23,24]. Furthermore, the study is supposed to provide valuable insight regarding usefulness of focused screening program in T2DM as effective therapies have evolved for HCV which may prevent complications caused by HCV in this subgroup.

The objective of this study was to determine the prevalence of HCV in patients with T2DM and to elucidate the presence of association between HCV and T2DM by comparing seropositivity rates in diabetic patients with a control group comprising of healthy volunteer blood donors from the same area. Data was also analyzed for the presence of difference among various groups with respect to various variables after dividing patients into two groups on basis of HCV infection status.

## Methods

### Participants

The study was carried out on a sample of 3000 consecutive persons with confirmed type 2 diabetes visiting Diabetes clinic at Nishtar Medical College Hospital, Multan for follow up. Subjects were excluded if they had type 1 diabetes, were transplant recipients, emergency cases or dialysis patients. A control group comprising of healthy blood donors were taken from the same hospital who visited the blood bank during the study period. Controls were excluded from the study if they had diabetes. Ethical clearance for the study was obtained from the Ethics Review Committee of hospital.

### Sample Collection and Antibody Detection

Sample collection was carried out using sterile needle and syringes which were transferred to the lab for analysis. 3 ml of peripheral blood was obtained via venepuncture for each subject. The blood was centrifuged and sera were stored in dry clean sterile containers at -20C prior to use. Serological analysis for the detection of anti-HCV antibodies was carried out using Accurate rapid immunochromatographic kits. Positive cases were later confirmed by using third generation ELISA kit (Labkit Chemelex, S.A., Barcelona, Spain) for qualitative detection of anti-HCV antibodies.

### Data Collection

After explaining the purpose of the study, written informed consent was taken from the participants before collecting data and taking samples. Data was collected using a structured questionnaire containing questions related to demographic and clinical characteristics of patients.

### Statistical Analysis

Data analysis was carried out using SPSS software. Descriptive analysis was performed and the results were expressed as means and percentages. Odds ratio (OR) and their respective 95% confidence intervals (CI) were calculated. Pearson chi-square test was used to determine the difference among various categories with respect to HCV seropositivity. A p value of <0.05 was considered statistically significant.

## Results

The study population comprised of 3000 patients with diabetes and 10,000 controls recruited from the blood bank. The mean age of diabetes patients was 48.19 ± 10.32 years and the mean duration of diabetes was 6.26 ± 5.50 years. Majority of patients with T2DM were female (55.7%) and in the age group 36-45 years (34.7%). Most of the cases were married (94.8%) and were from urban localities (71.8%). Family history of diabetes was present in only 19.5% of the participants with majority of participants having poor glycemic control (75.2%). Description of the study group is outlined in table 1. The controls were predominantly males with a median age of 27 years.

**Table 1:** Description of the study group (n = 3000)

	Diabetes Group	
**Variables**	**N**	**%**

**Age**		

≤35 Years	360	12.0

36-45 Years	1042	34.7

46-55 Years	933	31.1

>55 Years	665	22.2

**Gender**		

Male	1330	44.3

Female	1670	55.7

**Marital Status**		

Unarried	157	5.2

Married	2843	94.8

**Locality**		

Urban	2154	71.8

Rural	846	28.2

**Duration of Diabetes**		

1-5 Years	1653	55.1

6-10 Years	855	28.5

>11 Years	492	16.4

**Family History of Diabetes**		

Yes	2414	80.5

No	586	19.5

**Glycemic Control**		

Good	744	24.8

Bad	2256	75.2

Screening for the presence of anti-HCV antibody was positive in 906 (6.97%) patients in the entire study group. The seroprevalance was 13.7% in patients with T2DM as compared to the control group in whom prevalence rate was 4.9%. Analysis revealed that diabetic patients had significantly higher prevalence of HCV infection as compared to the control group (OR = 3.03, 95%CI = 2.64-3.48, p = 0.001).

The distribution of HCV infection in diabetic patients was then studied with respect to age, gender, marital status, locality, family history of diabetes, duration of diabetes and glycemic control. The results of analysis are presented in table 2. Patients older than 55 years, male patients, having duration of diabetes more than 11 years and the ones with good glycemic control were seen to be have significantly higher seropositive rates of HCV infection as compared to other groups in respective categories (p < 0.05). It was found that categories with respect to marital status, locality and family history of diabetes recorded no significant difference in seroprevalence among themselves (p > 0.05).

**Table 2:** Distribution of HCV pattern in diabetic patients (n = 3000)

	HCV Status				
				
Variables	Positive n(%)	Negative n(%)	OR	95% CI	P value
**Age**					.001
	
≤35 Years	38 (10.6%)	322 (89.4%)	-	-	
	
36-45 Years	103 (9.9%)	939 (90.1%)	0.93	0.63 - 1.38	
	
46-55 Years	101 (10.8%)	832 (89.2%)	1.03	0.69 - 1.53	
	
>55 Years	168 (25.3%)	497 (74.7%)	2.86	1.96 - 4.18	

**Gender**					.023
	
Male	203 (15.3%)	1127 (84.7%)	-	-	
	
Female	207 (12.4%)	1463 (87.6%)	0.79	0.64 - 0.97	

**Marital Status**					.075
	
Unmarried	14 (8.9%)	143 (91.1%)	-	-	
	
Married	396 (13.9%)	2447 (86.1%)	1.66	0.95 - 2.89	

**Locality**					.184
	
Urban	323 (15.0%)	1831 (85.9%)	-	-	
	
Rural	109 (12.9%)	737 (87.1%)	0.84	0.66 - 1.06	

**Duration of Diabetes**					.040
	
1-5 Years	231 (14.0%)	1422 (86.0%)	-	-	
	
6-10 Years	107 (12.5%)	748 (87.5%)	0.88	0.69 - 1.13	
	
>11 Years	90 (18.3%)	402 (81.7%)	1.38	1.05 - 1.80	

**Family History of Diabetes**					.143
	
Yes	319 (13.2%)	2095 (86.8%)	-	-	
	
No	91 (15.5%)	495 (84.5%)	1.21	0.94 - 1.55	

**Glycemic Control**					.001
	
Good	139 (18.7%)	605 (81.3%)	-	-	
	
Bad	268 (11.9%)	1988 (88.1%)	0.59	0.47 - 0.73	

## Discussion

In the present study, we found high prevalence of HCV infection in patients with T2DM as compared to the control group comprising of healthy volunteer blood donors (13.7% vs. 4.9%). The results of our study are in agreement with those of some studies conducted earlier in other countries [7,11,13,14,25,27,28,31]. The findings also agree with another study conducted earlier in the country albeit with a small sample use and no control group who recorded prevalence of 36% among diabetics [31]. This is thus the first study to the best of our knowledge in the country which employs a large sample comprising of consecutive patients compared with control group of consecutive blood donors. Furthermore, the prevalence of 4.9% in blood donors in our study is comparable to earlier studies from Pakistan [22]. The study thus establishes presence of T2DM as a risk factor for HCV infection in this region.

It was observed that older patients were more likely to have HCV infection as compared to those in the younger age groups [11,20,21,25,28]. The high seropositivity recorded in older group may be because of more parenteral exposures as compared to younger people and thus greater chances of transmission of infection.

Analysis of HCV seropositivity rates with respect to gender revealed that males had higher HCV infection rate of 15.3% as compared to females in whom prevalence rate of 12.4% was found. This agrees with the work of Caronia et al. who observed that male diabetics are more likely to contact HCV infections as compared to females [32]. A significant difference was also observed among patients with T2DM with respect to duration of diabetes. In relation to glycemic control, it was seen that patients with good glycemic control were significantly more at risk of having HCV infection as compared to those with poor glycemic control. This may be because of various confounding factors and warrants further investigation.

We did not observed any difference in the HCV distribution in study population with respect to marital status, place of residence of participants and family history of diabetes. Our findings regarding family history of diabetes differ from those of an earlier study from Nigeria [25]. The difference may be explained by the difference in proportion of patients with family history of diabetes (19.5% vs. 8.3%) and the difference in epidemiology of diabetes and HCV between the two countries.

The study has certain strength as well as same limitations. The study has relatively large sample size as compared to most of earlier studies and enrolled consecutive patients thus reducing recruitment bias and increasing generalizability of findings to a greater population. Secondly, the study used a control group for comparison of findings with the diabetic group. The control group was recruited from the same area as the cases. Furthermore, the study is first of its kind from the region and the first large scale controlled study from a country with a high number of patients with diabetes in which coinfection with HCV can have important implications. The study also has some limitations. We do not have the data on some of the potential confounding factors like exposure to risk factors such as blood transfusion. Moreover, we were not able to confirm the results of screening by using polymerase chain reaction to detect HCV RNA due to financial and technical constraints. Also we did not study liver enzyme levels in diabetic patients and controls.

The study has some important implications. The increased risk of HCV infection in patients with T2DM warrants screening diabetes person for HCV. Secondly, the study adds to the limited data on the subject available in this region and will help in increasing awareness regarding association of HCV and diabetes which will help in reducing morbidity and cost associated with this comorbidity in the long run. Prospective, multicentre studies are needed to establish temporal association, elucidate the reasons of association as well as the mechanism and determination of other aspects of the relationships.

## Conclusions

In conclusion, there is a significant association between Hepatitis C virus infection and type 2 diabetes in the region according to the findings of the present study. However, it remains to be seen whether diabetes is a risk factor for the HCV infection or vice versa. It is also evident that certain factors including older age, male gender, longer duration of diabetes and good glycemic control significantly increase the risk of having HCV infection which warrants special attention to patients with these risk factors. It is important that health care workers pay attention to prompt diagnosis and management of the condition in affected diabetic patients. Further investigation into the association of the two conditions is needed and may elucidate the temporal relationship and improved management strategies.

## List of Abbreviations

CLD: Chronic Liver Disease; HCC: Hepatocellular Carcinoma; HCV: Hepatitis C Virus Infection; ELISA: Enzyme Linked Immunosorbent Assay; T2DM: Type 2 Diabetes Mellitus

## Competing interests

The authors declare that they have no competing interests.

## Authors' contributions

NAJ conceived of the study and designed it, participated in data collection, coordinated the study, performed the statistical analysis and interpretation of data, prepared the draft of manuscript and reviewed it. MAS participated in design of the study, carried out data collection and participated in data analysis and reviewed the manuscript. RY participated in design of the study, carried out data collection and participated in data analysis. MH participated in data collection and analysis and reviewed the manuscript. NA participated in data collection and analysis. All the authors have read and approved the final manuscript.
